# Urine-NMR metabolomics for screening of advanced colorectal adenoma and early stage colorectal cancer

**DOI:** 10.1038/s41598-019-41216-y

**Published:** 2019-03-18

**Authors:** Eun Ran Kim, Hyuk Nam Kwon, Hoonsik Nam, Jae J. Kim, Sunghyouk Park, Young-Ho Kim

**Affiliations:** 1 Departments of Medicine, Samsung Medical Center, Sungkyunkwan University School of Medicine, Seoul, Korea; 2 College of Pharmacy, Natural Product Research Institute, Seoul National University, Sillim-dong, Gwanak-gu, Seoul, Korea; 3 Helsinki Institute of Life Science/Medicum, University of Helsinki, Helsinki, Finland

## Abstract

Although colorectal cancer (CRC) is considered one of the most preventable cancers, no non-invasive, accurate diagnostic tool to screen CRC exists. We explored the potential of urine nuclear magnetic resonance (NMR) metabolomics as a diagnostic tool for early detection of CRC, focusing on advanced adenoma and stage 0 CRC. Urine metabolomics profiles from patients with colorectal neoplasia (CRN; 36 advanced adenomas and 56 CRCs at various stages, n = 92) and healthy controls (normal, n = 156) were analyzed by NMR spectroscopy. Healthy and CRN groups were statistically discriminated using orthogonal projections to latent structure discriminant analysis (OPLS-DA). The class prediction model was validated by three-fold cross-validation. The advanced adenoma and stage 0 CRC were grouped together as pre-invasive CRN. The OPLS-DA score plot showed statistically significant discrimination between pre-invasive CRN as well as advanced CRC and healthy controls with a Q2 value of 0.746. In the prediction validation study, the sensitivity and specificity for diagnosing pre-invasive CRN were 96.2% and 95%, respectively. The grades predicted by the OPLS-DA model showed that the areas under the curve were 0.823 for taurine, 0.783 for alanine, and 0.842 for 3-aminoisobutyrate. In multiple receiver operating characteristics curve analyses, taurine, alanine, and 3-aminoisobutyrate were good discriminators for CRC patients. NMR-based urine metabolomics profiles significantly and accurately discriminate patients with pre-invasive CRN as well as advanced CRC from healthy individuals. Urine-NMR metabolomics has potential as a screening tool for accurate diagnosis of pre-invasive CRN.

## Introduction

Colorectal Cancer (CRC) is one of the most common causes of cancer-related deaths globally. The incidence of CRC is expected to exceed 2 million new cases by 2030, resulting in more than 1 million deaths. Rapid increases in CRC incidence and mortality have been described in many low/middle-income countries^1,2^. CRC development is characterized by very slow progression from adenoma to carcinoma due to the accumulation of various genetic and epigenetic mutations over decades^3^. A large proportion of CRC cases and deaths could be prevented by screening and early detection and removal of colorectal adenomas or early stage CRC^4,5^. Therefore, the development of reliable and non-invasive screening tools for early stage CRC and precancerous lesions, such as adenoma, is indispensable.

Colonoscopic screening and surveillance have a well-documented benefit in reducing the risk of CRC by direct removal of precancerous lesion and early detection of CRC^6,7^. However, the use of colonoscopy as a screening tool is limited because it is an invasive and unpleasant procedure that necessitates bowel preparation and sedation. Instead, the fecal occult blood test (FOBT) has been commonly used in the clinic. An unacceptably wide range or the lack of sensitivity and specificity of the FOBT has hampered its clinical application in CRC diagnosis, especially for precancerous lesions^8^. Tumor markers such as carcinoembryonic antigen (CEA) and carbohydrate antigen 19-9 (CA 19-9) are also commonly used in the clinic. However, these tumor markers cannot be used alone to screen for or diagnose CRC because their sensitivity is low. Blood levels of these components may rise due to benign diseases such as inflammatory bowel disease and pneumonia, and even in smokers. Thus, there are no non-invasive screening tools for detecting precancerous lesions, such as colorectal adenoma. Recently, endoscopic resection (ER) has been established as the therapeutic option for early CRC with no risk of lymph node metastasis^9^. Compared with surgery, ER advantages include reduced invasiveness, shorter hospital stay, and lower costs^10^. Therefore, the development of a low-cost, easy, and accurate diagnostic approach for the detection of advanced adenoma and early stage CRC would be essential for complete recovery of the patient and to reduce medical expenses.

Metabolomics is one of the emerging ‘omics’ studies used to investigate global or system-wide metabolic profiles. It provides a dynamic portrait of the metabolic status of living systems^11^. This approach has great potential in the diagnosis of various cancers using advanced analytic techniques and bioinformatics tools^12^, it has already been used for therapeutic monitoring and drug development^13,14^. A few metabolic markers are consistently found in CRC, but metabolic profiles of patients with early stage CRC including precancerous lesions remain poorly understood and warrant further investigation due to the non-invasive nature of the approach^15,16^. In the last decade, several metabolomics approaches were used to identify metabolic alterations in CRC using a variety of sample types including urine, tissue, serum and feces^17^, but only a few urinary metabolomics studies were published^18–21^. Nuclear magnetic resonance (NMR) spectroscopy is one of the major analytical techniques used in metabolomics research; it has several advantages including a relatively high degree of reproducibility, easy-to-identify metabolites, high throughput, and non-destructive sample treatment^22^. Although NMR-based metabolomics approach has many of these advantages, its usage in CRC has been limited; and only two NMR-metabolomics studies on CRC have recently been reported^23,24^.

Dykstra *et al*.^23^ conducted NMR-based urine metabolomics studies on CRC, but their research was focused on adverse events and responsiveness to chemotherapy in stage III and IV patients, search for personalized therapies. Wang *et al*.^24^ analyzed and compared urinary metabolic profile differences between early stage CRC (stages I and II) and healthy controls using an NMR-based metabolomics approach. The major finding of this research was the identification of stage I and II specific biomarkers indicating that several metabolisms including those from amino acids, glycolysis, the TCA cycle, the urea cycle, choline metabolism, and gut microflora metabolism were highly active in these stages.

It is widely accepted that most CRCs develop following the adenoma-carcinoma sequence, in which normal colorectal epithelium is transformed into benign neoplasm (adenoma) and, subsequently, into malignant neoplasm (invasive carcinoma) as a result of the accumulation of multiple genetic and epigenetic mutations. This implies that mutated genes, activated molecular pathways and, subsequently accumulated metabolites will be different at each stage of the adenoma-carcinoma sequence.

Here, we investigate the differences in the urine metabolic profiles of patients with colorectal neoplasia (CRN) including CRC and precancerous lesions, and healthy volunteers using an NMR-based urine metabolomics approach. In addition, we evaluate the applicability of this approach as a high sensitivity and specificity diagnostic tool especially for early detection of precancerous colorectal lesions.

## Results

### Participant characteristics

The characteristics of both patients and healthy controls are summarized in Table 1. The median age of patients with CRN (60 years; range 32–85) was older than that of healthy controls (52 years; range 22–76), and there were more males in the CRN group than in the control group (67.4% vs. 48.7%). Body mass indexes (BMI) of patients with CRN (23.56 kg/m^2^ in average; range 18.3–33.4) were slightly higher than those from healthy controls (23.0 kg/m^2^ in average; range 16.9–34.6). After patients underwent endoscopic resection or surgical resection for CRN; advanced adenoma was diagnosed in 36 patients; stage 0 CRC, in 24 patients; stage I CRC, in 8 patients; stage II CRC, in 7 patients; stage III CRC, in 13 patients; and stage IV CRC, in 4 patients. Cancers were classified according to the 7^th^ edition of the American joint Committee on Cancer (AJCC) cancer staging manual^25^.

**Table 1:** Characteristics of patients and healthy controls.

	Patients with colorectal neoplasia^a^ (n = 92)	Healthy controls (n = 156)
Age (years), median (range)	60(32–85)	52(22–76)
Gender, male (%)	62(67.4%)	76(48.7%)
BMI(kg/m^2^), median (range)	23.56 (18.3–33.4)	23.0(16.9–34.6)
Advanced adenoma	36	—
CRC^b^	56	—
TMN stage^c^		
0	24	
I	8	
II (IIA/IIB/IIC)	7	
III (IIIA/IIIB/IIIC)	13	
IV(IVA/IVB)	4	
Serum tumor marker (>cut off value^d^/total^e^)		
CEA	2/32	1/156
CA 19-9	2/32	1/156

^a^Colorectal neoplasia, including colorectal cancer and advanced adenoma.

^b^CRC, colorectal cancer.

^c^TNM stage, classified according to the American Joint Committee on Cancer (AJCC) 7^th^ edition.

^d^Cut off value of CEA, 5 ng/ml; Cut off value of CA19-9, 37 U/ml.

^e^Total number of patients with stage I to 4 CRC (n = 32 patients).

CEA and CA 19-9 levels for patients with stage I to IV CRC and for healthy controls were also assessed. Among patients with stage I to IV CRC, the level of CEA and CA 19-9 increased only in 2 individuals from each group. Therefore, the sensitivity and specificity of CEA and CA 19-9 were 6.2% and 99.3%, respectively.

### Discrimination and diagnosis of CRN and healthy control groups

**Figure 1:**
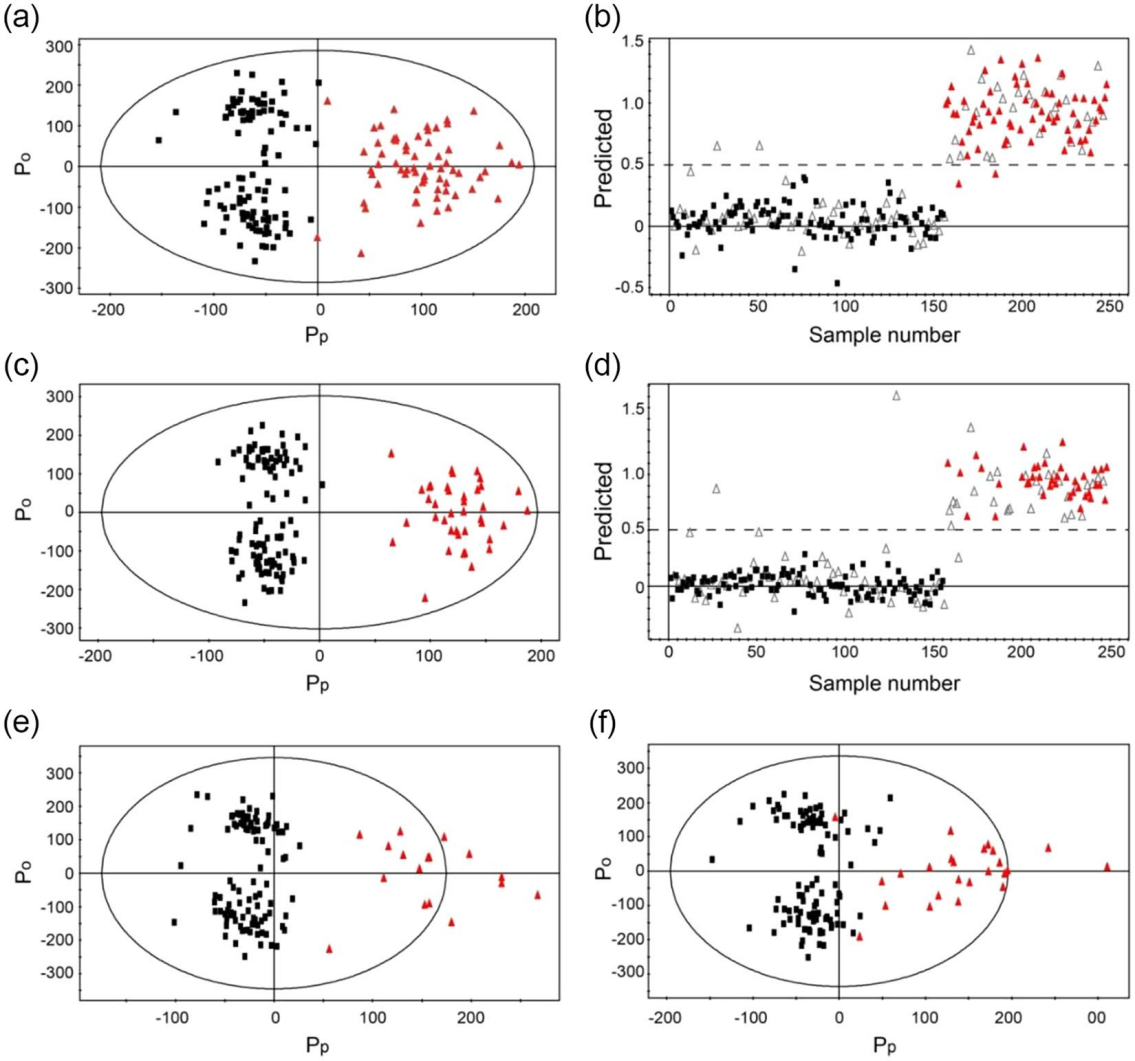
OPLS-DA score plots and prediction models for colorectal neoplasia and healthy controls. Models were obtained using one predictive (*P*_*p*_) and three orthogonal components (*P*_0_). The OPLS-DA models show good separation between (**a**) healthy controls vs. all colorectal neoplasia, (**b**) healthy controls vs. pre-invasive colorectal neoplasia, including advance adenoma and stage 0 CRC, (**c**) healthy controls vs. stage 0 CRC, and (**d**) healthy controls vs. advanced adenoma, (**e**) stage 0 CRC, and (**f**) advanced adenoma. The prediction model validation by three-fold cross-validation was based on the OPLS-DA (**b**,**d**). An *a priori* cut-off value of 0.5 was used to determine the prediction result. Black boxes: healthy control group; Red triangles: colorectal neoplasia group; Open triangles: unknown samples.

We analyzed all the NMR spectroscopy-based urine metabolic profiles and performed multivariate statistical analyses to discriminate between CRN patients and healthy controls. OPLS-DA was applied to our data because it can discriminate between groups even in the presence of high structured noise or confounding factors. As indicated in Fig. 1a, the OPLS-DA score plot was created using one predictive (*P*_*p*_) and three orthogonal components (*P*_0_); it shows a significant discrimination between the CRN and the healthy control groups with an R2 value (overall goodness of fit) of 0.864 and a Q2 value (overall cross-validation coefficient) of 0.714 (Table 2). The prediction model was built using the same number of predictive and orthogonal components as in the OPLS-DA score plot, after determining an *a priori* cut-off value of 0.5, resulting in 96.2% specificity and 100% sensitivity for pre-invasive CRN diagnosis (Fig. 1b and Table 2).

**Table 2:** Diagnostic analysis of the urine-NMR metabolomics samples in detecting colorectal neoplasia.

		Healthy control (n = 156)		CRN^a^ (n = 92)		Model R2/Q2		Specificity	Sensitivity
		Model	Validation	Model	Validation				
All CRN^*^	Samples	104	52	62	30	R2	0.864	96.2%	100%
	Prediction	50/52		30/30		Q2	0.714		
Pre-invasive CRN^b^	Samples	104	52	40	20	R2	0.845	96.2%	95%
	Prediction	50/52		19/20		Q2	0.732		
Stage 0 CRC^c^	Samples	104	52	17	7	R2	0.830	98.1%	100%
	Prediction	51/52		7/7		Q2	0.644		
Advanced adenoma	Samples	104	52	24	12	R2	0.727	100%	75%
	Prediction	52/52		9/12		Q2	0.634		

^a^CRN, colorectal neoplasia including colorectal cancer and advanced adenoma.

^b^Pre-invasive CRN, stage 0 colorectal cancer and advanced adenoma.

^c^CRC, Colorectal cancer.

### Early stage CRN diagnosis

For the CRN diagnosis, we used patient samples from all CRC stages including advanced adenoma and stage 0 to IV CRCs; discrimination between CRN and healthy control groups is shown as two different data concentration groups in the plot (Fig. 1a). As the majority of CRN patients were diagnosed as very early CRC stages including advanced adenoma (n = 36) and stage 0 CRC (n = 24), our results suggest that this approach would be effective on early stage diagnosis. Therefore, we moved onto earlier stage diagnosis using only advanced adenoma and stage 0 patient samples. When advanced adenoma and stage 0 CRC were grouped as pre-invasive CRN, which is an indication of endoscopic resection as first-line therapy, the OPLS-DA score plot revealed statistically significant discrimination between pre-invasive CRN and healthy control groups (Fig. 1c), and presented similar sensitivity and specificity for diagnosing pre-invasive CRN of 96.2% and 95%, respectively (Fig. 1d and Table 2). When stage 0 CRC (Fig. 1e) and advanced adenoma (Fig. 1f) were analyzed independently, they were statistically well discriminated from the healthy control group with over 75% sensitivity and over 98% specificity. According to the cross-validation function of the SIMCA-P software, we obtained reasonable R2 and Q2 values over 0.72 and over 0.63, respectively. Diagnostic analysis results are presented in Table 2.

### Metabolic differences between colorectal neoplastic lesions and healthy controls

**Figure 2:**
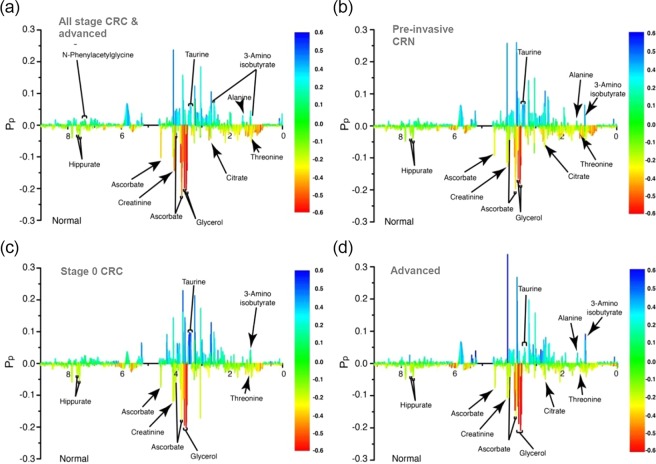
Identification of metabolites contributing to colorectal neoplasia (CRN). Variable contributions from statistical total correlation spectroscopy (S-TOCSY) show the model coefficients for each NMR variable. (**a**) Healthy controls vs. all colorectal neoplasia, (**b**) Healthy controls vs. pre-invasive colorectal neoplasia including advance adenoma and stage 0 CRC, (**c**) Healthy controls vs. stage 0 CRC, and (**d**) Healthy controls vs. advanced adenoma. The color scale based on the value of *P*(*coor*)*p*, according to weight is used as a discriminator between two groups. *P*_*p*_ represents the modeled covariant. Signals, color coded metabolites, that significantly discriminate between the two groups were annotated on the model coefficient plot.

**Figure 3:**
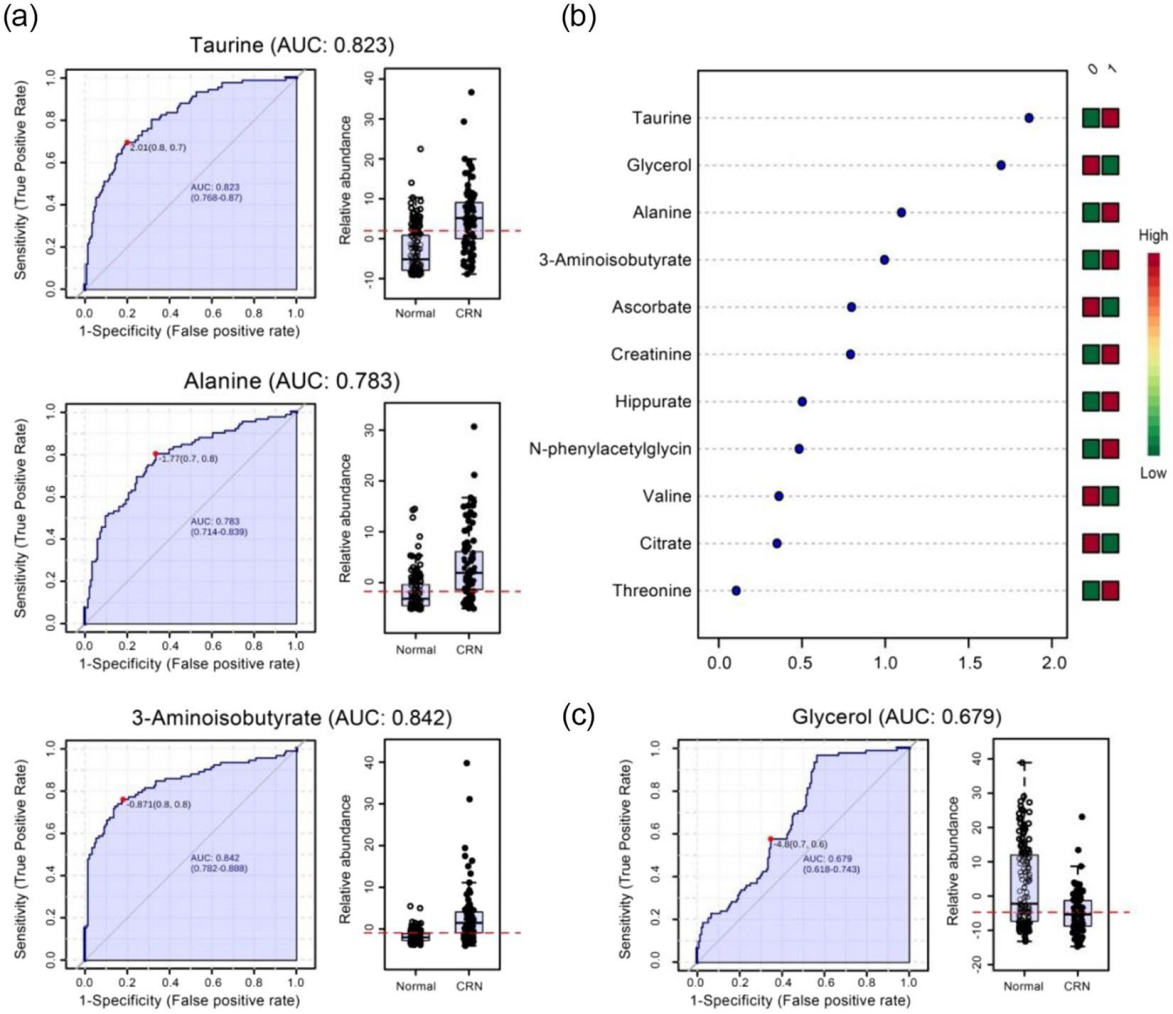
ROC analysis of contributing metabolites for discrimination. (**a**) OPLS-DA based ROC curve analysis for diagnosis of pre-invasive colorectal neoplasia (CRN). Taurine, alanine, 3-aminoisobutyrate showed very high AUC scores. (**b**) Multiple ROC curve analysis for 11 metabolites contributing for discrimination. The color scale on the right shows whether each metabolite concentration level was increased or decreased in CRN. (**c**) ROC curves of glycerol showing high ranking on the multiple ROC curve analysis, but poor sensitivity and specificity.

Major contributing metabolites for group separation were identified by statistical total correlation spectroscopy (S-TOCSY; Fig. 2). Representative metabolites were slightly different according to each CRC stage analyzed; also relative amounts of taurine, alanine, 3-aminoisobutyrate, and valine were greater in CRN samples than in healthy controls, where relative amounts of threonine, glycerol, hippurate, ascorbate, creatinine and citrate were greater in healthy controls than in CRN groups (Tables 3 and 4). To evaluate the importance of discriminating metabolites, the receiver operating characteristic (ROC) curve analysis was applied to our data. Predicted importance by the OPLS-DA model showed that the areas under the curve (AUCs) were 0.823 for taurine, 0.783 for alanine and 0.842 for 3-aminoisobutyrate (Fig. 3a). In multiple ROC analyses, several metabolites including taurine, glycerol, alanine and 3-aminoisobutyrate were implicated as good discriminators for CRN patients (Fig. 3b). However, we excluded glycerol as a concerning metabolite, although it seemed as a secondly important metabolite in multiple ROC analysis, because of its poor AUC value (Fig. 3c).

**Table 3:** Representative markers according to CRN stages.

**Healthy control**	All stage	Threonine	Glycerol	Hippurate	Ascorbate	Creatinine	Citrate
	Pre-invasive CRN						
	Stage 0						
	Advanced adenoma						Citrate
**Colon cancer**	All stage	Valine	3-Aminoisobutyrate	Taurine	Alanine	N-phenyl-acetylglycine	
	Pre-invasive CRN						
	Stage 0						
	Advanced adenoma				Alanine

**Table 4:** Marker identification for early detection of colorectal neoplasia.

no	Metabolites	Chemical shift (ppm)	p-value	Changes
1	**3-Aminoisobutyrate**	1.18(d)	5.83 × 10^−06^	▲
2	**Alanine**	1.48(d),	1.21 × 10^−03^	▲
3	Ascorbate	3.75(m), 4.53(m)	6.81 × 10^−10^	▽
4	Citrate	2.54(d), 2.71(d)	3.66 × 10^−05^	▽
5	Creatinine	3.05(s), 4.07(s)	1.30 × 10^−2^	▽
6	Glycerol	3.58(m), 3.66(m), 3.78(m),	5.22 × 10^−32^	▽
7	Hippurate	3.98(d), 7.56(t), 7.64(t), 7.83(m)	4.53 × 10^−04^	▽
8	**Taurine**	3.27(t), 3.45(t)	2.86 × 10^−04^	▲
9	Threonine	1.31(d), 3.58(d)	7.03 × 10^−03^	▽
10	Urea	5.80(s)	7.26 × 10^−04^	▲
11	Valine	0.99(d)	8.35 × 10^−07^	▲

### Diagnostic accuracy of the study

Although the population used in our discrimination models have low prevalence, varying from 11.9 to 36.6%, most of their sensitivities and specificities were over 95%, except for the advanced adenoma population which had a 75% sensitivity (Table 5). However, sensitivity and specificity do not provide information about the probability of a test^26,27^. Therefore, we also calculated the predictive values and likelihood ratio (LR) from our data. Interestingly, the positive predictive values (PPVs), as well as the negative predictive values (NPVs) presented high scores, over 87.6% and up to 100% for different patients’ cancer stages and prevalence. Moreover, when the LR values are >10 and <0.1, it means that there is strong evidence to rule in or rule out diagnosis in most circumstances^28^; our diagnostic results showed LR values >25 and <0.05 which are in close correspondence with this rationale. Diagnostic accuracy parameters are summarized in Table 5.

**Table 5:** Diagnostic accuracy values.

	Prevalence	Sensitivity	Specificity	PPV^a^	NPV^b^	(+) LR^c^	(−) LR^d^
All CRN	36.6%	100%	96.2%	93.8%	100%	26.0	0.00
Pre-invasive CRN	27.8%	95%	96.2%	90.5%	98.0%	24.7	0.05
Stage 0 CRN	11.9%	100%	98.1%	87.6%	100%	52.0	0.00
Advanced adenoma	18.8%	75%	100%	100%	94.6%	N/A	0.25

^a^PPV, positive predictive value; (sensitivity * prevalence)/[sensitivity * prevalence + (1 − specificity) * (1 − prevalence)].

^b^NPV, negative predictive value; [specificity * (1 − prevalence)]/[(1 − sensitivity) * prevalence + specificity * (1 − prevalence)].

^c^(+) LR, positive Likelihood Ratio; sensitivity/(1 − specificity).

^d^(−) LR, negative Likelihood Ratio; (1 − sensitivity)/specificity.

## Discussion

The metabolomics approach for the discrimination and diagnosis of CRC patients has been applied to a variety of human samples. The majority of those studies used either tissue or blood samples, but only a few involved feces or fecal water extractions. Wang *et al*.^19^ reported metabolomics profiling of tissue samples, using in ^1^H NMR, from a large cohort of rectal cancer patients and healthy controls. The authors made an excellent separation and demonstrated the existence of distinguishing metabolites among different stages of rectal cancer tissues and healthy controls. Another group analyzed metabolites in intact tumor samples and samples of adjacent mucosa obtained from 26 patients undergoing surgical resection for CRC using high-resolution magic angle spinning NMR^20^.

They reported that tumor-adjacent mucosa (10 cm from tumor margin) harbors unique metabolic field changes that distinguish tumors according to T- and N-stage with a high predictive capability, as good as tumor tissue itself.

Although metabolomics profiling using tissue samples provides good discrimination between CRC and healthy controls, it is difficult to use as a screening test for early detection of CRC because the test sample is usually obtained by invasive methods. Therefore, searching for useful and easy to obtain test samples, several researchers analyzed the serum or fecal metabolomics profiles from CRC patients. In one study, the metabolomics approach was used to search for potential diagnostic biomarkers in the serum of 30 patients surgically treated for CRC^21^. Of all the analyzed metabolites, the concentrations of only 6 were significantly increased or decreased compared to those from control samples. Supervised predictive models allowed a separation of 93.5% of CRC patients from healthy controls using these metabolites. However, they were not able to classify and analyze the results according to CRC stages.

NMR was also used to profile the serum metabolome in metastatic CRC (mCRC) patients and to determine whether a disease signature is predictive of overall survival (OS)^22^. In the training set, NMR metabolomics profiling was able to discriminate patients with mCRC with a cross-validated accuracy of 100%. Also, patients with short or long OS could be identified by proton NMR (^1^H-NMR) profiling with an accuracy of 78.5%. Lin *et al*. determined fecal metabolites of 68 CRC patients (Stage I/II = 20, Stage III = 25, and Stage IV = 23) and 32 healthy controls using ^1^H NMR, establishing the differences between the CRC stages. They reported that even early CRC stages (stage I/II) were clearly distinguished from healthy controls based on their metabolomics profiles^23^. These results showed the potential utility of metabolomics as a diagnostic marker in CRC. However, more data are needed for fecal metabolomics profiling before this approach can be adopted with confidence. Especially, there is a lack of data about advanced adenoma or stage 0 CRC which is an indication of less invasive endoscopic treatment. Furthermore, although obtaining fecal samples is non-invasive, it is a less pleasant experience compared with urine collection.

Recently, several NMR-based urine metabolomics studies have been reported for CRC. One of these studies only analyzed samples from patients with stage III and IV CRC to investigate adverse events and responsiveness to chemotherapy^23^. In contrast, the purpose of our study is to develop a diagnostic tool using an NMR-based urine metabolomics approach based on a better understanding of the metabolic profile of patients with early CRC. The purpose of Dykstra’s research is much related to the development of a personalized medicine treatment based on metabolic profiles, rather than to the development of diagnostic methods for early CRC patients. Therefore, it has a different character from the final goal we pursue in this study.

In other urinary metabolomics study that used samples from 20 CRC patients and 14 healthy controls, a panel of metabolic markers composed of citrate, hippurate, p-cresol, 2-aminobutyrate, myristate, putrescine, and kynurenate was able to discriminate CRC subjects from their healthy counterparts^15^. However, the early stage CRC (stages I and II) group comprised only 8 patients, and none had precancerous lesions. From our perspective, the most recent research conducted by Wang *et al*.^24^ is particularly interesting and relevant to our study as it analyzed samples from early I/II CRC patients. They showed urinary metabolic differences between early stage colorectal cancer and healthy controls and provided 16 potential CRC biomarkers related to several key metabolic pathways.

In contrast, the present study contains more than 65% early stage CRN patients, including 39% with advanced adenoma (n = 36) and 26% with stage 0 CRC (n = 24). Since CRC is slowly aggravated by adenoma-carcinoma sequence for a long time, early diagnosis is essential to elevate the survival rate and to ease the treatment for the patients^29^. Therefore, this study, which analyzes patients at an earlier stage CRC than previous studies, will be highly valuable for the clinic; it presents strong data indicating the potential of urine metabolomics as a screening tool for early stage CRC.

To date, our study is the first and the largest NMR-based urine metabolomics study of CRN patients including precancerous lesions such as advanced adenomas and stage 0 CRC. For developing a diagnostic model, the sample type and the way it is collected are very important for the patient, both convenience and preference. In this regard, even though urine is a very attractive biofluid for clinical purposes, including diagnosis or prognosis prediction, it has not been used often for CRC metabolomics studies. This is reflected in a relative scarcity of evidence of change in urine metabolites in CRC. However, more than 300 metabolites are detectable in urine and their concentration levels may be used as signature of systemic diseases.

Our current study shows that urine metabolomics has potential for CRN diagnosis with extremely high sensitivity and specificity. Surprisingly, our results also show a strong diagnostic power for patients with precancerous lesions and early stage CRN. Additionally, the statistical parameters showed highly reliable R2 and Q2 values, over 0.72 and over 0.63, respectively. A large discrepancy between R2 and Q2 value indicates an overfitting of the model, but our diagnostic result presented theoretically ideal R2 and Q2 values^30^. According to the SIMCA users’ guide, the Q2 value is an estimate of the predictive ability of the model, calculated by cross-validation, and a Q2 value over 0.65 together with an R2 value over 0.78 would be indicative of a good model^31^.

Although sensitivity and specificity are widely used diagnostic accuracy parameters, their high dependence on prevalence is a well known limitation^32^. Therefore, the use of predictive value and likelihood ratio reflecting the prevalence is very important to overcome these vulnerabilities^33^. The PPV represents the probability of having the disease after a positive test result, whereas the NPV is the probability of not having the disease after a negative test result^34,35^. However, the predictive values have one critical limitation; they could only be applied when the clinical prevalence is identical. So, the use of an alternative diagnostic parameter, less dependent on the prevalence, is necessary; thus we adopted the LR, a combination of sensitivity and specificity. The LR can be used in conjunction with disease prevalence to estimate an individual patient’s probability of having a disease^31,32^. In other words, the LR is less dependent on the prevalence because it has the advantage of giving equal weights to the sensitivity and specificity. Our diagnostic results showed ideal LR points.

CRC is a common malignant tumor and has become a major public health concern. Despite improved treatment approaches done over the past decade, the outcomes or survival of patients with advanced disease have remained depressingly poor, and treatment costs have increased^24^. CRC is sporadic in 90% of patients and most CRC cases develop from a preclinical benign precursor, adenoma to invasive cancer over the span of years. The characteristics of CRC including high incidence, protracted and treatable preclinical phase, high cost of treatment and correlation of mortality with disease stage are well-suit for population screening^24,25^. FOBT is a well-known CRC screening method. The advantages of this method when compared for example with colonoscopy are its simplicity and cost-effectiveness. However, the sensitivity of FOBT is low and its validity remains debatable. Also, it has been demonstrated that the highest validity rate is needed for successful CRC screening^26^. The purpose of screening strategies is to save cost through early disease detection and treatment. Therefore, screening test for CRC must be able to detect adenoma or early stage CRC. For this reason, out finding is important and meaningful.

In this study, we identified several metabolites useful as CRN discrimination factors including taurine, alanine, 3-aminoisobutyrate (BAIB), valine, threonine, glycerol, hippurate, ascorbate, creatinine and citrate. Multiple ROC curve analyses were performed to evaluate the importance of discriminating metabolites; we chose taurine, alanine, and BAIB as key contributing metabolic markers based on them. Although glycerol presented as a secondly important metabolite from multiple ROC analyses, its poor AUC value of 0.679 is a critical reason to exclude it from the key marker selection. Interestingly, the three selected metabolites are similar in structure and correlated with metabolic pathways. BAIB homeostasis could also be correlated with congenital abnormality of the colorectal systems, and its concentration level is increased in gastric cancer tissues^27,28^. Still, direct evidence is lacking concerning the role of BAIB in various cancers. However, several reports have indicated that BAIB has important effects on energy metabolism including pyrimidine metabolism, beta oxidation, and glucose homeostasis. Cancer cells need and consume excessive energy for their fast and aggressive growth and metastasis, so energy supplementation must be completed in any way. We suggest that the identified CRC representative metabolites taurine, alanine, and BAIB could be part of the energy supplementation intermediates. Taurine and alanine could be energy sources by themselves, whereas BAIB might control energy metabolism, which should be upregulated in cancer patients.

Current NMR-based urine metabolomics results showed high sensitivities and specificities for discriminating colorectal neoplasia.

Interestingly, regardless of low prevalence, all diagnostic parameters including sensitivity, specificity, predictive value, and likelihood ratio are good enough represented.

In conclusion, NMR-based untargeted urine metabolomics approach significantly differentiates between patients with CRN and healthy controls. Our results also indicated that urinary metabolic profiles have strong potential as screening tools for accurate diagnosis of pre-invasive CRN.

## Methods

### Participants

A total of 92 patients with CRN including CRC or advanced adenoma were enrolled from July 2013 to April 2016. They underwent endoscopic resection or surgical resection for CRN. In this study, advanced adenoma was defined tubular adenoma, tubulovillous adenoma, villous adenoma or serrated adenoma with a size ≥1 cm. Stages 0 to IV CRC were determined according to the American joint Committee on Cancer (AJCC) 7^th^ edition. Stage 0 CRC was defined either as cancer cells confined within the intraepithelial or mucosal lamina propria with no extension into submucosa, or adenoma demonstrating a high grade dysplasia. Advanced adenoma and stage 0 CRC were grouped as pre-invasive CRN.

The healthy control group (normal) comprised 156 healthy subjects who underwent health screening. Healthy controls had no detected abnormalities, neither in blood tests, nor in endoscopic examination, diagnostic imaging, nor medical interview.

Patients with prior treatment including chemotherapy or surgery, serious complications including active bleeding or obstruction, decompensated liver cirrhosis, active hepatitis, collagen disease, severe uncontrolled diabetes mellitus (HbA1c > 7.5%), chronic renal failure (glomerular filtration rate <30 ml/min/1.73 m^2^ or on dialysis), and pre-existing carcinoma at other sites were excluded.

This study was approved by the institutional review board of Samsung Medical Center. All the treatment methods were carried out in accordance with the approved guideline. Informed consent was obtained from all participants.

### Sample collection, preparation and NMR experiments

The first morning urine samples (3 to 5mL) were collected and centrifuged at 3000 rpm for 15 minutes at 4 °C. Supernatants were transferred to frozen tubes and stored at –80 °C until processing. Frozen urine samples were thawed at room temperature and immediately centrifuged at 15,000 rpm at 4 °C for 20 min. After centrifugation, 50 µL of phosphate buffer (1.5 M K_2_HPO_4_ + 1.5 Na_2_HPO_4_, pH 7.4) were added to 500 µL of supernatant and incubated at room temperature for 10 min. Incubated mixtures were centrifuged before 450 µL of supernatant were blended together with a 50 µL 0.25% trimethylsilanepropionic acid (TSP) solution in deuterium oxide. Finally, the whole 500 µL of this mixture were transferred to a 5 mm standard NMR tube. All the proton NMR spectra were acquired using a 500 MHz NMR spectrometer (Bruker Biospin, Avance 500, Billerica, MA, USA). Experimental parameters and data acquisition processes were basically similar to those previously reported^29^.

### Data processing and statistical analysis

Proton NMR spectral data were manually processed by Fourier transformation, phase correction, baseline correction, referencing, and normalized against the internal standard 0.025% TSP signal. All processed data were binned to a 0.0092 ppm width by an in-house Perl script, except for the water signal region (4.6–5.2 ppm), and numerically transformed. Transformed data were analyzed with statistical software including SIMCA-P version 11.0 (Umetrics, Umeå, Sweden), OrigionPro 8 (OriginLab Corporation, Northampton, MA, USA), and R (The R Foundation for Statistical Computing, Vienna, Austria). For statistical analysis, all the imported data were normalized as mean-centered scaling using the Pareto scaling algorithm in SIMCA-P software. Healthy control and colorectal neoplastic lesion groups were statistically discriminated using orthogonal projections to latent structure discriminant analysis (OPLS-DA).

### Metabolites identification and representative marker selection

For the representative metabolites identification, all proton NMR signals were referenced against an internal standard TSP signal and identified using the Chenomx NMR database (Spectral Database, Edmonton, Alberta, Canada). In brief, all the experimental proton NMR signals from patients were fit into the Chenomx database by shifting under appropriate pH ranges. Identified metabolites were exported as numeric values and the area under curve (AUC) was analyzed using an open source receiver operating characteristics (ROC) curve analysis tool^30^.

### Validation of prediction models for different stages

The class prediction model was validated as previously described^31^. Briefly, one-third of the samples of each group (52 healthy control and 30 CRN samples) were blinded without any group information and the rest of the samples (104 healthy control and 62 CRN samples) were used for prediction model construction; 0.5 was used as an *a priori* cut-off value for evaluating the prediction model^32^, and the sensitivity and specificity were calculated. Positive predictive values (PPVs) were calculated by the following formula: (sensitivity * prevalence)/[sensitivity * prevalence + (1 − specificity) * (1 − prevalence)]; while negative predictive values (NPVs) were calculated by: [specificity * (1 − prevalence)]/[(1 − sensitivity) * prevalence + specificity * (1 − prevalence)]; positive likelihood ratio (+LR), by: sensitivity/(1 − specificity); and negative likelihood ratio (-LR), by: (1 − sensitivity)/specificity. The same approach was applied to pre-invasive CRN prediction validation.

## References

[1] Arnold M (2017). Global patterns and trends in colorectal cancer incidence and mortality. Gut.

[2] Center MM, Jemal A, Smith RA, Ward E (2009). Worldwide variations in colorectal cancer. CA Cancer J Clin.

[3] Brenner H, Stock C, Hoffmeister M (2015). Colorectal cancer screening: the time to act is now. BMC Med..

[4] Shaukat A (2013). Long-term mortality after screening for colorectal cancer. N Engl J Med.

[5] Brenner H, Stock C, Hoffmeister M (2014). Effect of screening sigmoidoscopy and screening colonoscopy on colorectal cancer incidence and mortality: systematic review and meta-analysis of randomised controlled trials and observational studies. BMJ.

[6] Doubeni CA (2016). Effectiveness of screening colonoscopy in reducing the risk of death from right and left colon cancer: a large community-based study. Gut.

[7] Lee JL (2017). Determining the optimal surveillance interval after a colonoscopic polypectomy for the Korean population?. Intestinal research.

[8] Levin B (2008). Screening and surveillance for the early detection of colorectal cancer and adenomatous polyps, 2008: a joint guideline from the American Cancer Society, the US Multi-Society Task Force on Colorectal Cancer, and the American College of Radiology. CA Cancer J Clin.

[9] Watanabe T (2012). Japanese Society for Cancer of the Colon and Rectum (JSCCR) guidelines 2010 for the treatment of colorectal cancer. Int J Clin Oncol.

[10] Heo J (2014). Endoscopic resection as the first-line treatment for early colorectal cancer: comparison with surgery. Surg Endosc.

[11] Goodacre R, Vaidyanathan S, Dunn WB, Harrigan GG, Kell DB (2004). Metabolomics by numbers: acquiring and understanding global metabolite data. Trends Biotechnol..

[12] Spratlin JL, Serkova NJ, Eckhardt SG (2009). Clinical applications of metabolomics in oncology: a review. Clin. Cancer Res..

[13] Puchades-Carrasco L, Pineda-Lucena A (2017). Metabolomics Applications in Precision Medicine: An Oncological Perspective. Curr Top Med Chem.

[14] Wei R (2011). Metabolomics and its practical value in pharmaceutical industry. Curr Drug Metab.

[15] Wang H, Tso VK, Slupsky CM, Fedorak RN (2010). Metabolomics and detection of colorectal cancer in humans: a systematic review. Future Oncol..

[16] Armitage EG, Barbas C (2014). Metabolomics in cancer biomarker discovery: current trends and future perspectives. J. Pharm. Biomed. Anal..

[17] Zhang A (2014). Metabolomics in diagnosis and biomarker discovery of colorectal cancer. Cancer Lett..

[18] Ma YL (2009). Ultra-high performance liquid chromatography-mass spectrometry for the metabolomic analysis of urine in colorectal cancer. Dig. Dis. Sci..

[19] Qiu Y (2010). Urinary metabonomic study on colorectal cancer. J Proteome Res.

[20] Cheng Y (2012). Distinct urinary metabolic profile of human colorectal cancer. J. Proteome Res..

[21] Chen, J.-L. *et al*. Urine metabolite profiling of human colorectal cancer by capillary electrophoresis mass spectrometry based on MRB. *Gastroenterology research and practice* **2012** (2012). 10.1155/2012/125890 PMC3518074 23243419

[22] Markley JL (2017). The future of NMR-based metabolomics. Curr. Opin. Biotechnol..

[23] Dykstra MA (2017). Urine metabolomics as a predictor of patient tolerance and response to adjuvant chemotherapy in colorectal cancer. Molecular and clinical oncology.

[24] Wang Z (2017). NMR-based metabolomic techniques identify potential urinary biomarkers for early colorectal cancer detection. Oncotarget.

[25] Edge SB, Compton CC (2010). The American Joint Committee on Cancer: the 7th edition of the AJCC cancer staging manual and the future of TNM. Ann. Surg. Oncol..

[26] Altman DG, Bland JM (1994). Diagnostic tests. 1: Sensitivity and specificity. BMJ: British Medical Journal.

[27] Akobeng AK (2007). Understanding diagnostic tests 1: sensitivity, specificity and predictive values. Acta Paediatr..

[28] Davidson M (2002). The interpretation of diagnostic tests: A primer for physiotherapists. Aust. J. Physiother..

[29] Haggar FA, Boushey RP (2009). Colorectal cancer epidemiology: incidence, mortality, survival, and risk factors. Clin. Colon Rectal Surg..

[30] Triba MN (2015). PLS/OPLS models in metabolomics: the impact of permutation of dataset rows on the K-fold cross-validation quality parameters. Mol. Biosyst..

[31] Umetrics, A. User guide to SIMCA-P+12. *Kinnelon: Umetrics Inc* (2008).

[32] Leeflang, M. M., Rutjes, A. W., Reitsma, J. B., Hooft, L. & Bossuyt, P. M. Variation of a test’s sensitivity and specificity with disease prevalence. *Can. Med. Assoc. J*., cmaj. 121286 (2013). 10.1503/cmaj.121286 PMC3735771 23798453

[33] Bossuyt PM (2003). The STARD statement for reporting studies of diagnostic accuracy: explanation and elaboration. Ann. Intern. Med..

[34] Altman DG, Bland JM (1994). Statistics Notes: Diagnostic tests 2: predictive values. BMJ.

[35] Van Stralen KJ (2009). Diagnostic methods I: sensitivity, specificity, and other measures of accuracy. Kidney Int..

